# Breaking the Cycle: Enhancing Cardiovascular Health in the Elderly Through Group Exercise

**DOI:** 10.3390/life15020206

**Published:** 2025-01-29

**Authors:** Lovorka Bilajac, Mihaela Marinović Glavić, Zulle Kristijan, Bilobrk Matea, Denis Juraga, Ana Jelaković, Tomislav Rukavina, Vanja Vasiljev, Bojan Jelaković

**Affiliations:** 1 Department of Social Medicine and Epidemiology, Faculty of Medicine, University of Rijeka, 51000 Rijeka, Croatia; lovorka.bilajac@uniri.hr (L.B.); denis.juraga@medri.uniri.hr (D.J.); anajelakovic9@gmail.com (A.J.); tomislav.rukavina@uniri.hr (T.R.); vanjav@medri.uniri.hr (V.V.); 2 Faculty of Health Studies, University of Rijeka, 51000 Rijeka, Croatia; kristijan.zulle@uniri.hr; 3 Teaching Institute of Public Health of Primorje—Gorski Kotar County, 51000 Rijeka, Croatia; bilobrk5@gmail.com; 4 Department of Nephrology, Hypertension, Dialysis and Transplantation, University Hospital Center Zagreb, 10000 Zagreb, Croatia; jelakovicbojan@gmail.com; 5 School of Medicine, University of Zagreb, 10000 Zagreb, Croatia

**Keywords:** cardiovascular risks, elderly, hypertension, physical activity, waist-to-hip ratio (WHtR)

## Abstract

The global increase in aging populations underscores the urgency of addressing cardio–kidney metabolic health indicators, particularly among sedentary elderly individuals. This study investigates the impact of an 8-month structured group exercise program on cardiovascular health indicators among 320 women aged 60 and older living independently in Rijeka. Participants engaged in biweekly sessions designed to improve mobility, balance, and strength. Key metrics, including blood pressure (BP), body mass index (BMI), waist-to-hip (WHR) and waist-to-height ratio (WHtR), and hand grip strength, were measured before and after the intervention. Results revealed significant reductions in systolic blood pressure (mean −3.4 mmHg) and pulse pressure among hypertensive participants, highlighting improved cardiovascular function. BP control significantly improved (7.2%), and 19% of untreated hypertensive subjects at the start become normotensive at the end of follow-up. Although BMI changes were minimal, WHtR improvements indicated reductions in central obesity and muscle fat redistribution. Hand grip strength increased significantly on both arms, correlating with physical capacity. The results underline the benefits of group training for improving health even in the elderly population through an organized exercise program. While these preliminary results demonstrate promising health improvements, further research with longer follow-up and inclusion of diverse participant groups is recommended to validate these outcomes and refine intervention strategies.

## 1. Introduction

As the global population ages, maintaining the cardiovascular health of older people has become an increasingly important public health challenge [1]. Cardiovascular disease (CVD) remains the leading cause of morbidity and mortality in older adults and is often exacerbated by physical inactivity, a common lifestyle pattern in this population. Prolonged inactivity not only accelerates the decline in cardiovascular function but also contributes to a number of other health problems, including obesity, diabetes, and hypertension [2]. The prevalence of overweight and obesity in adults and the elderly remains high in 2024, as evidenced by anthropometric measurements. Body mass index (BMI), waist-to-hip ratio (WHR), and waist-to-height ratio (WHtR) are used as predictive factors for hypertension [3,4,5]. Obesity, especially abdominal obesity, plays an important role in the development of hypertension, and the use of these indicators can help identify patients at higher risk. Early intervention in these individuals, which includes non-pharmacological methods of monitoring hypertension, optimizes the possibilities for secondary prevention measures.

Despite the clear risks associated with a sedentary lifestyle, many older people find it difficult to switch to more active habits. Factors such as physical limitations, fear of injury, and lack of motivation often prevent them from exercising regularly. However, emerging evidence suggests that participation in structured exercise groups can significantly improve cardiovascular health and general well-being in older people, even those who lead a predominantly sedentary lifestyle [6].

Group exercise programs provide not only physical activity but also social support, which is essential for sustained participation. These programs can be particularly effective in promoting a gradual transition from sedentary behavior to an active lifestyle adapted to the specific needs of the elderly population [7]. By fostering a sense of community and shared purpose, exercise groups can help overcome the psychological and physical barriers that often prevent older adults from engaging in regular physical activity [8,9]. A review of recent studies and analysis of group exercise interventions will be used to highlight the potential benefits of these programs and provide insight into their effective implementation to improve cardiovascular outcomes in this vulnerable population [9,10].

Given these challenges, this study specifically investigates the impact of group physical activity on key health indicators in older women living in the community. Our aim was to analyze the impact of group physical activity on reducing cardiovascular health risks by measuring blood pressure, measures of obesity, and hand grip strength in elderly women living in their own households.

## 2. Materials and Methods

This is a pre–post interventional study investigating the effects of a structured group exercise program on cardiovascular and physical functioning in elderly women.

A total of 320 participants aged 60 years and over were included in this study. The inclusion criteria were participants aged 60 years or older, mobile, living independently in the city of Rijeka, with a diagnosed cardiovascular disease (I00-I99)—diseases of the blood vessel system. Exclusion criteria were persons with terminal malignant disease, those with dementia or disability (paralysis or amputations), and those recovering from surgery or acute illness.

**Figure 1:**
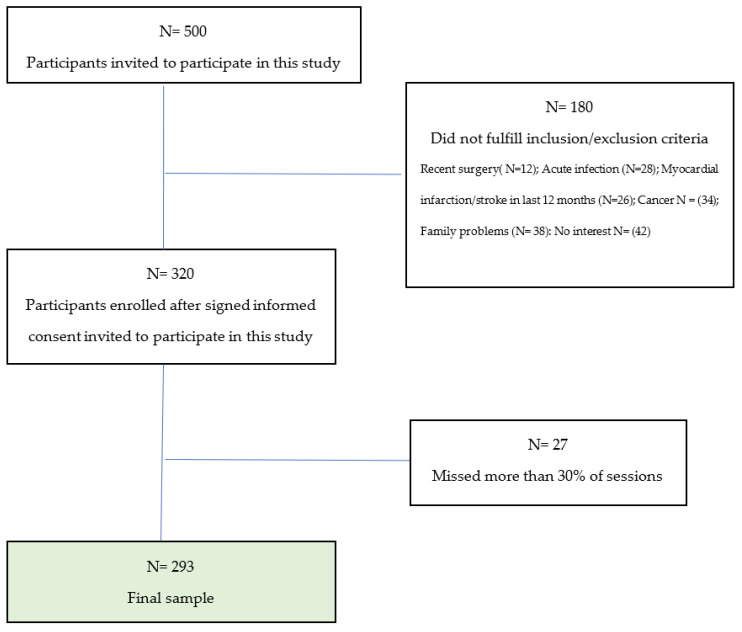
Final number of participants included in this study.

The patronage nurses selected the participants in the first year according to the inclusion criteria, after which they applied for the intervention themselves. In addition, participants who missed more than 30% of the sessions were excluded (Figure 1). This study was conducted from December 2021 to May 2024.

The research included measurements at two time points and a structured physical activity intervention.

### 2.1. Structured Physical Activity

The intervention phase lasted 8 months and included regular group exercises twice a week for one hour. The participants were divided into smaller exercise groups (25–30 participants per group). The exercise program was designed and led by trained physiotherapy students supervised by older experts. Exercises were specifically adapted to the needs of older people. A training plan was created consisting of structured physical activities adapted to the age and health status of the participants. The training plan included warm-up exercise (light activities to prepare the body for exercise, 10 min); the main part of the exercise aimed to increase range of motion, improve balance, coordination, and proprioception, and strengthen muscles (35–40 min); and a cool down and stretching part (which includes relaxation and stretching exercises to gradually lower heart rate and improve flexibility, 10–15 min) (Table 1). Physical activity was defined as participation in a standardized, structured 8-month group exercise program. The program was specifically tailored to the needs of older people and included functional exercises to improve strength, balance, coordination, and flexibility. All participants attended at least 70% of the sessions.

### 2.2. Measurements

Measurements were taken at the beginning of the program and after 8 months of participation. All measurements were taken by trained personnel following standardized protocols to ensure consistency and accuracy. Measurements included height, weight, blood pressure (BP), hand grip strength, and waist and hip circumference.

Body weight was measured using a digital medical scale (Omron Viva Smart Scale, Kyoto, Japan), and the participants were dressed only in light clothes and without shoes. Height was measured using a SECA 214 stadiometer (Seca GmbH & Co. KG, Hamburg, Germany), with participants standing upright and barefoot. Body mass index (BMI) was calculated as the ratio of body weight (in kilograms) to the square of height (in meters). BMI is a widely used indicator for categorizing people as underweight, normal weight, overweight, and obese based on set cut-off points. Body mass index was calculated as follows: underweight (BMI < 18.5 kg/m^2^); normal weight (18.5 ≤ BMI < 25 kg/m^2^); overweight (25 ≤ BMI < 30 kg/m^2^); and obese (BMI ≥ 30 kg/m^2^) [11].

Waist and hip circumference were measured using a non-stretchable anthropometric tape, with participants standing upright. Waist circumference was measured on bare skin at the narrowest point between the lowest rib and the iliac crest. Hip circumference was measured at the widest part of the buttocks. Both measurements were recorded to the nearest millimeter, and duplicate measurements were taken to ensure accuracy. Waist circumference, waist-to-height ratio, and waist-to-hip ratio were used to assess abdominal obesity. These indices were used to assess the participants’ risk of developing hypertension, cardiovascular disease, and other metabolic disorders.

Waist-to-hip ratio (WHR) was calculated by dividing the waist circumference (in centimeters) by the hip circumference (in centimeters). WHR is often used as a measure of abdominal obesity, with higher values indicating a greater risk of metabolic and cardiovascular disease. WHR values for older adults, focusing on health risks, are as follows: less than 0.85: low risk (healthy fat distribution); 0.85–0.90: moderate risk (potentially increased risk of health problems); and 0.90 or higher: high risk (significant risk of cardiovascular diseases and other issues related to visceral fat) [5]. Waist-to-height ratio (WHtR) was calculated by dividing waist circumference (in centimeters) by height (in centimeters). Waist-to-height ratio (WHtR) values for older adults, based on available research and general health guidelines, are as follows: less than 0.40: low risk (healthy fat distribution, lower risk of cardiovascular and metabolic diseases); 0.4–0.55: healthy range; 0.55–0.65: increased risk (potentially increased risk of cardiovascular and metabolic health issues; indicates central obesity); and 0.65 or higher: high risk (significant risk of cardiovascular diseases and other health complications associated with central obesity) [5]. WHtR is considered a useful predictor of cardiometabolic risk, with a ratio above 0.5 generally indicating increased health risks.

Before measuring the BP, the participant sat quietly with the back of the chair and did not speak for at least 5 min. Both feet were flat on the floor. Blood pressure was measured on both arms using an oscillometric device (Omron M7 sphygmomanometer, Dalian, China). The hand being measured should have been relaxed on the table, and the participant was not allowed to hold the hand up. During the measurement, the device also recorded the heart rate [12].

Hand grip strength was measured using a Yamar hydraulic hand dynamometer. Measurements were performed with the participant standing, shoulders neutrally rotated and adducted, the elbow flexed at 90°, and the forearm in a neutral position. Each subject performed two measurements, and the mean value for both arms was used for further statistical analysis [13].

### 2.3. Statistical Analysis

Body mass index (BMI), waist-to-hip ratio (WHR), and waist-to-height ratio (WHtR) were calculated using standard formulas based on the anthropometric measurements collected during the study. The statistical analysis was performed to evaluate the impact of the physical activity on anthropometric and cardiovascular health indicators. The data were collected at two time points: before the 8-month group program and after. The collected data were analyzed using IBM SPSS Statistics 27.0.0.0. (IBM Corp., Armonk, NY, USA). Before the appropriate statistical test was used, normality of data was assessed using the Kolmogorov–Smirnov test.

Descriptive statistics, including means, standard deviations, medians, and interquartile ranges, were calculated for continuous variables. Categorical variables were summarized using frequencies and percentages. Baseline and follow-up values were compared to assess initial differences and trends over time. To compare the pre- and post-intervention values, for normally distributed continuous variables, the Paired Sample *t*-test was used, and for non-normally distributed variables, the Wilcoxon Signed-Rank Test was used.

Physical activity was defined as a standardized intervention. Changes in hand grip strength, measured as a continuous variable, were analyzed before and after the intervention. Pearson’s correlation coefficient (r) was used to assess the strength and direction of improvement in hand grip strength before (T0) and after (T1) the intervention. Correlations were interpreted as follows: weak (<0.3), moderate (0.3–0.5), and strong (≥0.5). Statistical significance was set at a *p*-value of <0.05 for all analyses.

Ethical aspects: This study is being conducted in accordance with the Declaration of Helsinki and has been approved by the local ethics committee. All participants have signed an informed consent and participated voluntarily in this study.

## 3. Results

Baseline characteristics of participants and measurements are presented in Table 2. The mean age of participants was 69.9 ± 6.

Blood pressure values are presented in Table 3. Systolic BP was significantly lower at the follow-up visit in the whole group (Λ = −3.4 mmHg) and in hypertensive subjects (Λ = −4.4 mmHg). In normotensive subjects, we observed no changes or just a slight drop (Λ = −0.5 mmHg). There was no significant difference in diastolic BP. Importantly, we found pulse pressure to be significantly improved in hypertensive subjects (Λ = −2.9 mmHg) and modestly in normotensive women (Λ = −2.9 mmHg). Results on prevalence, treatment, and control of hypertensive subjects are shown in Table 4. Control of treated hypertensive subjects substantially improved at the end of the follow-up period (for 7.2%), but the difference did not reach statistical significance. In the untreated hypertensive subjects, four of them become normotensive.

**Figure 2:**
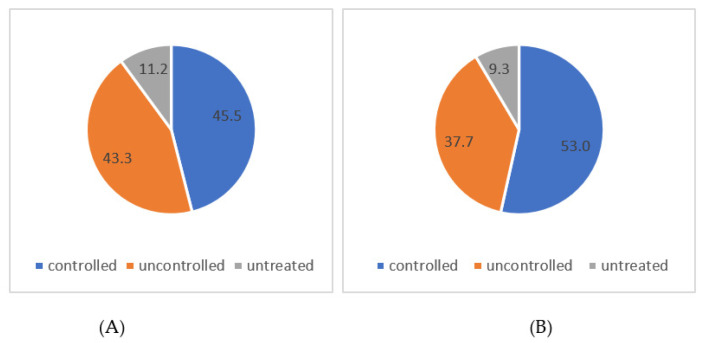
Distribution of controlled, uncontrolled, and untreated hypertensive subjects at the first visit (**A**) and at the follow-up visit (**B**).

HT participants were divided into two major groups: treated (N = 166) and untreated (N = 21) HT participants. Untreated participants were those with HT who were not taking any medication. In addition, treated HT participants were divided into controlled and uncontrolled HT groups. Participants with uncontrolled hypertension were those with diagnosed HT and prescribed medication but not reaching target values. Of the 187 (63.8%) participants with HT, 88.7% were treated 51.2 % of them was under control after the measurements at the first visit. At the follow-up visit, the percentage of controlled participants increased to 58.4%. We noted a slight decrease in the percentage of untreated participants (11.2% vs. 9.3%) (Table 4, Figure 2).

The body mass index (BMI) values of the participants at baseline and at the end of the follow-up period are shown in Table 5. The difference in BMI values was statistically significant (*p* < 0.001). However, the minimal change in mean BMI suggests that it may not reflect a clinically relevant change in BMI. Nevertheless, we also found that 2.4% participants who were obese became overweight, which, with the previous finding, might indicate that a more beneficial effect on BMI could be achieved in a longer period of physical activity.

Analysis of the waist-to-hip ratio (WHR) and waist-to-height ratio (WHtR) measurements in participants is presented in Table 6. These measurements serve as important indicators of body composition and are associated with metabolic health risks.

At baseline (T0), data were collected from 293 participants distributed across the WHR risk categories as follows: low risk: 75 participants (25.6%); moderate risk: 97 participants (33.1%); and high risk: 121 participants (41.3%).

In the follow-up measurement (T1), in which 270 participants took part, the distribution was as follows: low risk: 63 participants (23.3%); moderate risk: 103 participants (38.2%); and high risk: 104 participants (38.5%).

We failed to find a statistically significant difference in the distribution of WHR risk categories during the study period. Although there was a slight increase in the proportion of participants in the moderate-risk category and a slight decrease in both the low- and high-risk categories, these changes were not significant. This result suggests a stable distribution of WHR risk among participants, with minimal shifts between categories from baseline to follow-up.

On the contrary, the WHtR data showed a statistically significant positive change (*p* = 0.002) (Table 6).

**Figure 3:**
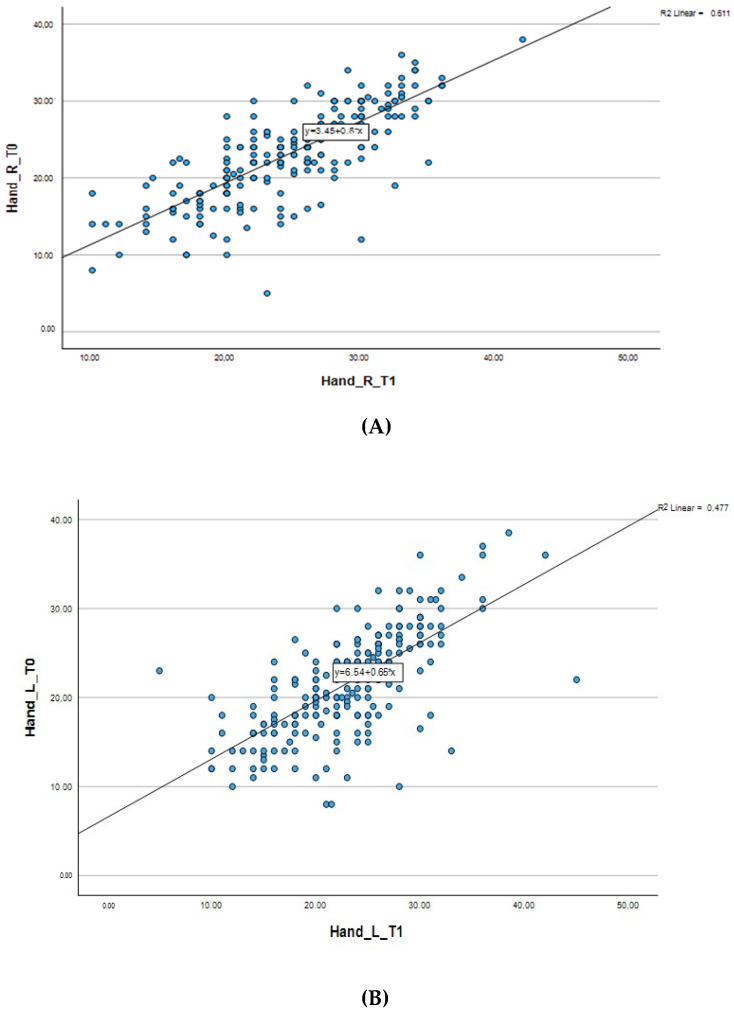
Correlation of right-hand grip strength (**A**) and left-hand grip strength (**B**) between baseline and follow-up measurements.

The relationship between hand grip strength measurements at baseline (T0) and follow-up measurements (T1) during 8 months of structured physical activity intervention is shown in Table 7 and Figure 3.

For the right hand, the average hand grip strength at the first measurement was 23.16 kg, while it increased to 24.66 kg after participation in the physical activity. Similarly, for the left hand, which was the non-dominant hand for most people, the average hand grip strength was 21.69 kg at the first measurement and increased to 23.15 kg at the second measurement. The observed changes in both hands indicate a consistent improvement in muscle strength.

The strong positive correlation between T0 and T1 of hand grip strength, indicated by the Pearson correlation coefficient (r = 0.78 for the right hand and r = 0.69 for the left hand), suggests that participation in the structured exercise program was strongly associated with improvements in muscle strength. Both correlations were statistically significant (*p* < 0.001), highlighting the functional improvements in hand grip strength as a result of the intervention.

## 4. Discussion

Aging is a known risk factor for the development of cardiovascular disease (CVD), which is often associated with increased obesity and excess body weight. Identifying risk factors at an early stage is crucial to mitigate potential negative health consequences. Among the various prevention strategies, regular physical activity has been shown to be one of the most important measures, with significant health benefits even in older people. Monitoring anthropometric indicators in older people allows early detection of health risks and thus supports targeted interventions.

In this study, we obtained very interesting and valuable preliminary results after only eight months of organized physical activity in elderly women living alone. Most of them were hypertensive and mostly had isolated systolic AH, and only one-third were normal weight.

### 4.1. Effects of Regular Group Exercise on Blood Pressure and Hypertension

The first important result was significant systolic BP reduction in women with HT, while BP reduction was not observed in normotensive subjects. BP reduction was consequently related to PP reduction. The increase in systolic BP and pulse pressure (PP) values after the age of 50 is much greater in women than in men [14]. PP is a surrogate measure of large arterial stiffness. Both systolic BP reduction and PP reduction are directly reflected in CV risk reduction in general. Systolic BP was on target; thus, HT control was accomplished in 15% treated, previously uncontrolled HT subjects. This is in line with other studies in postmenopausal women engaged in structural organized physical activity [15]. In a meta-analysis by Xi et al., the aerobic combined resistance exercise significantly decreased systolic blood pressure (SBP) and diastolic blood pressure (DBP) by 0.81 mmHg (95% CI, −1.34 to −0.28) and 0.62 mmHg (95% CI, −1.11 to −0.14), respectively, during an average time of 12 weeks. The effect of physical activity was most pronounced in overweight women > 60 years of age [16]. Another meta-analysis found much larger decrease in BP, PP, and PWV. The overall decrease in BP was −6.19 mmHg [95%CI −9.24 to −3.15, *p* < 0.0001], −3.56 mmHg [95%CI (−6.10, −1.03), *p* = 0.006), and −8.52 [95%CI (−16.27, −0.76), *p* = 0.03] for systolic BP and diastolic BP and PP, respectively [17]. Most data in the reviewed literature show that baseline BP levels had a large impact on the effect of exercise training, similar to our results [18].

### 4.2. Effects of Regular Group Exercise on Obesity Indices

Body mass index (BMI) is a widely accepted measure used by the World Health Organization (WHO) to classify individuals as underweight, normal weight, overweight, or obese [11]. Due to its simplicity, it offers a practical and straightforward approach to population-level monitoring. According to WHO standards for the European population, a BMI between 18.5 and 24.9 kg/m^2^ is within the healthy range [11]. In addition, BMI does not take into account fat distribution patterns, which are critical to understanding metabolic health risks [5,19]. In our study, BMI change was statistically significant but clinically irrelevant. A statistically significant shift was observed for WHtR as a marker of body fat redistribution and increase in lean body mass in our subjects. In study of high-functioning men and women (age of 70–79), all-cause mortality over 12 years of follow-up was associated only with WHR, not BMI nor waist circumference. WHR is far better tool for assessing obesity risk in the elderly, presumably because the physical changes that are part of the aging process alter the body proportions on which BMI is based [20].

Waist-to-hip ratio (WHR) and waist-to-height ratio (WHtR) provide complementary insights by indicating central adiposity, which is more strongly associated with cardiometabolic risks [21,22]. The WHR assesses the proportional distribution of fat between the waist and hips, while the WHtR provides a simple measure by comparing waist circumference to height. These indices are valuable for detecting abdominal obesity, which is often associated with a higher risk of cardiovascular and metabolic disease. In clinical and research contexts, combining BMI with measures of central adiposity such as WHR and WHtR provides a more comprehensive assessment of body composition and associated health risks. This multifaceted approach increases accuracy in identifying individuals at greater risk for health-related conditions [23]. Waist-to-height ratio (WHtR) is another measure used to assess health risks, particularly to identify abdominal obesity. Recent studies have shown that WHtR is the most accurate indicator for screening women with hypertension [3,24,25]. In contrast to WHR, WHtR provides a more comprehensive measure, as it takes into account body size, which may be a key factor in assessing fat distribution and overall health risks. The WHR and WHtR analyses provide a more detailed insight into participants’ body composition and associated health risks. WHR, an indicator of central obesity and cardiovascular risks, showed no statistically significant change from T0 to T1 (*p* = 0.545). The proportion of participants in the WHR categories “low”, “moderate”, and “high risk” remained largely stable, with only minor fluctuations. This stability may indicate that participants maintained an even distribution of fat around the waist and hips, which did not improve significantly during the study period. In the Women Initiative Study, WHtR > 0.5, WC > 88 cm, WHR ≥ 0.85, BMI > 25, and weight loss ≥ 5 kg were each directly associated with greater risk of CV, independent of other known CV risk factors. They also observed significant interactions of WHtR with WHR or WC but not with BMI in predicting CV risk during 17 years of follow-up. While waist circumference (WC), WHR, and WHtR capture specific changes in abdominal fat, WC is less sensitive to CV risk, particularly in women with the same abdominal fat but different heights [26].

On the other hand, the WHtR data showed a statistically significant shift with a *p*-value of 0.002, indicating a positive change in abdominal fat distribution and the associated health risk. Specifically, the percentage of participants in the healthy WHtR range increased from 27.7% at baseline to 33.3% at follow-up, while the percentage in the high-risk category decreased from 15.0% to 11.9%. This improvement in WHtR reflects a reduction in central obesity, which is particularly important for metabolic health and cardiovascular risk reduction. These results suggest that WHtR may be a more sensitive measure than BMI or WHR to track positive changes in body composition. This change could be due to several factors, including possible improvements in lifestyle, adjustments in medication use, or a reduction in the severity of hypertension in some participants. Group exercise may increase healthy lifespan and provide a better quality of life [7,27]. However, the observed increase in participants with hypertension who were not taking medication raises concerns about potential gaps in adherence or access to ongoing blood pressure treatment.

### 4.3. Effects of Regular Group Exercise on Hand Grip Strength

Finally, we observed a statistically significant increase in hand grip strength after the 8-month physical activity intervention. A surrogate measurement of overall muscular strength is a predictor of all-cause mortality in the elderly and may serve as a convenient prognostic tool for mortality risk among elderly people [28]. Hand grip strength has been shown to be a valuable indicator of cardiovascular risk, as it is related to overall muscle health, functional capacity, and physical fitness. Research has consistently associated reduced hand grip strength with an increased risk of cardiovascular disease (CVD) [29]. It is an indicator of muscle mass and metabolic health, as weaker hand grip strength reflects reduced muscle mass and decreased functional capacity, which are often associated with a higher risk of metabolic disorders such as type 2 diabetes, hypertension, and dyslipidemia [30].

The association with cardiovascular risk factors means that individuals with decreased hand grip strength often have elevated blood pressure, higher body mass index (BMI), unfavorable lipid profiles, and insulin resistance—all critical factors for the development of cardiovascular disease [31]. Hand grip strength serves as a screening and risk assessment tool, as it is a simple, non-invasive, and cost-effective method of assessing cardiovascular risk, particularly in older adults and individuals with pre-existing risk factors such as obesity or physical inactivity [32]. The demonstrated relationship between hand grip strength and cardiovascular health emphasizes the importance of maintaining muscle strength through regular physical activity. Strengthening exercises and an active lifestyle can play an important role in reducing overall cardiovascular risk and promoting healthier aging [33].

### 4.4. Limitations and Strengths of the Study

The most important strength of our research is its longevity, which is among the longest in the literature for such a group of subjects. Our cohort was very well defined, and we included both hypertensive and normotensive subjects, and the intervention in the form of group exercise lasted 8 months.

There are several limitations. This is a single-center report, and more data could be obtained if more centers were included. We did not include women living in nursing homes, but our goal was to analyze the effect of group exercise in women living in their own households. Our plan is to conduct a similar study with elderly people living in nursing homes and compare the results. We included only Caucasians and only urban women, so our results should not be extrapolated to other races and rural residents. Future research should include male participants and focus on metrics such as waist-to-height ratio, which has been shown to be more sensitive than BMI.

## 5. Conclusions

Eight months of organized physical activity in older women resulted in significant health improvements, including lower blood pressure values, better hypertension control, lower pulse pressure, redistribution of fat tissue, and increased arm strength. Preliminary results suggest improvements and efficacy, but larger studies and longer follow-up are needed to confirm the findings. In addition, the long-term efforts in health literacy could have influenced the outcomes. Group exercise programs should be preferred for their benefits to physical, mental, and social health.

## Figures and Tables

**Table 1:** Structure of exercise (low-impact aerobic exercise).

Part	Purpose	Time
Warm-up exercise	Light activities to prepare the body (heart, muscles, and joints) for training (shoulder rolls, arm swings, brisk walking)	10 min
Main part of the exercise	Increasing the range of movement; improving balance, coordination, and proprioception; and strengthening the muscles (with elastic bands, exercise sticks, small 0.5 kg weights, small balls)	35–40 min
Cool down and stretching part	Relaxation and stretching exercises to gradually lower the heart rate and improve flexibility (shoulder stretch, seated knees to chest, lateral overhead stretch, triceps stretch)	10–15 min

**Table 2:** Baseline characteristics of participants.

	Total (293)
Age, years	69.9 ± 6
Weight, kg	71.9 ± 10.23
BMI, kg/m^2^	27.21 ± 3.62
Waist circumference, cm	94.5 ± 9.5
Hip circumference, cm	107.2 ± 7.98
Systolic blood pressure, mmHg	133.4 ± 18.89
Diastolic blood pressure, mmHg	78.5 ± 9.26
Hand grip, right hand, kg	23.46 ± 5.83
Hand grip, left hand, kg	21.94 ± 5.69

**Table 3:** Blood pressure and pulse pressure values in hypertensive and normotensive subjects and difference between first and follow-up visit (Λ).

	N/%	Systolic Blood Pressure			Diastolic Blood Pressure			Pulse Pressure		
		First Visit	Follow-Up Visit	Λ	First Visit	Follow-Up Visit	Λ	First Visit	Follow-Up Visit	Λ
Whole group	293/100	135.0	131.6	−3.4	78.9	78.4	−0.5	56.1	53.2	−2.9
Hypertensives	192/65.5	139.7	135.3	−4.4	80.5	79.3	−1.2	58.9	56.0	−2.9
Normotensives	101/34.5	120.5	120.0	−0.5	73.8	75.6	+1.8	46.2	44.4	−1.8

**Table 4:** Prevalence, treatment, and control of hypertension at the first visit and at the end of the follow-up period.

	First Visit		Follow-Up Visit	
	N	%	N	%
HT subjects total	187	63.8	183	62.5
HT subjects treated	166	88.7	166	88.7
Controlled HT	85	51.2	97	58.4
Uncontrolled HT	81	48.8	69	41.6
Untreated	21	11.2	17	9.3

**Table 5:** Body mass index (BMI) values at baseline (T0) and follow-up (T1) measurements.

	T0 Measurement (*n* = 293)			T1 Measurement (*n* = 267)			*p*
	$\bar{X}$ (σ)	Min–Max	Std. Dev	$\bar{X}$ (σ)	Min–Max	Std. Dev	
BMI (kg/m^2^)	27.21 (3.62)	19.88–39.97	3.62	26.80 (3.58)	19.50–38.10	3.58	<0.001

kg = kilogram; m = meter; T0 = baseline measurement; T1 = follow-up measurement; *n* = number of participants; $\bar{X}$ = mean value; σ = standard deviation; Min = minimum; Max = maximum. *p* = *p*-value; level of marginal significance within a statistical hypothesis test, representing the probability of the occurrence of a given event.

**Table 6:** Participants’ waist-to-hip and waist-to-height ratios at baseline (T0) and follow-up (T1) measurements.

	T0 Measurement (*n* = 293)	T1 Measurement (*n* = 270)	*p*
WAIST-TO-HIP RATIO (WHR)	*n* (%)	*n* (%)	0.545
Low risk	75 (25.6)	63 (23.3)	
Moderate risk	97 (33.1)	103 (38.2)	
High risk	121 (41.3)	104 (38.5)	
Mean	0.88	0.054	
Std. Deviation	0.056	0.054	
WAIST-TO-HEIGHT RATIO (WHtR)			h
Low risk	0 (0.0)	0 (0.0)	0.002
Healthy range	81 (27.7)	90 (33.3)	
Increased risk	168 (57.3)	148 (54.8)	
High risk	44 (15.0)	32 (11.9)	
Mean	0.58	0.57	
Std. Dev.	0.06	0.05	

A statistical hypothesis test, representing the probability of the occurrence of a given event; WHR = waist-to-hip ratio; WHtR = waist-to-height ratio; T0 = baseline measurement; T1 = follow-up measurement; n = number of participants; % = percentage. *p* = *p*-value; level of marginal significance within *p* < 0.05.

**Table 7:** Correlation of hand grip strength during the 8-month exercise program.

	T0	T1	r	*p* *
Right hand	23.16	24.66	0.78	<0.001
Left hand	21.69	23.15	0.69	<0.001

* Correlation is significant at the 0.01 level (2-tailed).

## Data Availability

The collected data are presented in the paper and are available upon request from the corresponding author for any additional inquiries.
